# Increased physical activity severely induces osteoarthritic changes in knee joints with papain induced sulfate-glycosaminoglycan depleted cartilage

**DOI:** 10.1186/ar4461

**Published:** 2014-01-29

**Authors:** Michiel Siebelt, Harald C Groen, Stuart J Koelewijn, Erik de Blois, Marjan Sandker, Jan H Waarsing, Cristina Müller, Gerjo JVM van Osch, Marion de Jong, Harrie Weinans

**Affiliations:** 1 Department of Orthopedics, Erasmus Medical Center, P.O. Box 2040, 3000 CA Rotterdam, The Netherlands; 2 Department of Nuclear Medicine, Erasmus Medical Center, Rotterdam, The Netherlands; 3 Center for Radiopharmaceutical Sciences PSI-ETH-USZ, Paul Scherrer Institute, Villigen-PSI, Villigen, Switzerland; 4 Department of Otorhinolaryngology, Erasmus Medical Center, Rotterdam, The Netherlands; 5 Department of Biomechanical Engineering, Delft University of Technology, Delft, The Netherlands; 6 Department of Orthopaedics, UMC Utrecht, Utrecht, The Netherlands; 7 Department of Rheumatology, UMC Utrecht, Utrecht, The Netherlands

## Abstract

**Introduction:**

Articular cartilage needs sulfated-glycosaminoglycans (sGAGs) to withstand high pressures while mechanically loaded. Chondrocyte sGAG synthesis is regulated by exposure to compressive forces. Moderate physical exercise is known to improve cartilage sGAG content and might protect against osteoarthritis (OA). This study investigated whether rat knee joints with sGAG depleted articular cartilage through papain injections might benefit from moderate exercise, or whether this increases the susceptibility for cartilage degeneration.

**Methods:**

sGAGs were depleted from cartilage through intraarticular papain injections in the left knee joints of 40 Wistar rats; their contralateral joints served as healthy controls. Of the 40 rats included in the study, 20 rats remained sedentary, and the other 20 were subjected to a moderately intense running protocol. Animals were longitudinally monitored for 12 weeks with *in vivo* micro-computed tomography (μCT) to measure subchondral bone changes and single-photon emission computed tomography (SPECT)/CT to determine synovial macrophage activation. Articular cartilage was analyzed at 6 and 12 weeks with *ex vivo* contrast-enhanced μCT and histology to measure sGAG content and cartilage thickness.

**Results:**

All outcome measures were unaffected by moderate exercise in healthy control joints of running animals compared with healthy control joints of sedentary animals. Papain injections in sedentary animals resulted in severe sGAG-depleted cartilage, slight loss of subchondral cortical bone, increased macrophage activation, and osteophyte formation. In running animals, papain-induced sGAG-depleted cartilage showed increased cartilage matrix degradation, sclerotic bone formation, increased macrophage activation, and more osteophyte formation.

**Conclusions:**

Moderate exercise enhanced OA progression in papain-injected joints and did not protect against development of the disease. This was not restricted to more-extensive cartilage damage, but also resulted in pronounced subchondral sclerosis, synovial macrophage activation, and osteophyte formation.

## Introduction

Articular cartilage is evolutionarily designed to facilitate joint motion. Cartilage extracellular matrix (ECM) is composed of a collagen matrix in which chondrocytes reside. These cells produce high concentrations of sulfated-glycosaminoglycans (sGAGs), which contain negatively charged sulfate groups that set the cartilage fixed-charged density. Because of this fixed-charged density, large amounts of cations and water enter the cartilage ECM, expanding the collagen network and creating a high hydrostatic pressure. Cartilage daily endures high-peak mechanical loading, including shear, compression, and tension (contact) stresses. During physical activity, it is estimated that compressive stresses can increase to 10 to 20 MPa [1]. The high internal hydrostatic pressure allows articular cartilage to absorb these stresses and facilitates the dissipation and distribution of external forces during joint mobilization [2-4].

The amounts and types of external mechanical loading are important factors that regulate development and long-term maintenance of cartilage. This is because chondrocytes closely regulate sGAG levels dependent on the level of physical activity [5]. For example, in hamsters, a sedentary lifestyle is known to reduce cartilage sGAG content, whereas daily exercise prevents this loss [6]. Galois *et al.*[7] investigated whether different running intensities influenced osteoarthritis (OA) progression and found that moderate running protected against OA development in anterior cruciate ligament-transected knee joints. In another experiment with mono-iodoacetate (MIA)-induced OA, exercise also prevented cartilage damage. MIA inhibits glyceraldehyde-3-phosphate dehydrogenase activity, resulting in chondrocyte apoptosis and sGAG loss [8]. When MIA-injected rats were subjected to treadmill running, the superficial and intermediate areas of the joint showed a better preservation of sGAG content [9]. These studies support the idea that a mild biomechanical stressor on cartilage enhances chondrocyte ability to sustain sGAG levels and protect cartilage against OA onset.

Kiviranta *et al.*[10] also showed that moderate running augments sGAGs in articular cartilage of beagle dogs. However, later, they found that a strenuous exercise protocol induced marked sGAG depletion from superficial cartilage zones [11]. Since then, strenuous exercise has been shown to reduce chondrocyte metabolism and sGAG synthesis [12]. Besides inhibited sGAG production during strenuous exercise, chondrocytes also start actively to deplete sGAG from cartilage, which is facilitated through increased matrix metalloproteinase-13 (MMP-13) production [13]. So cartilage loading through strenuous running seems to elicit an imbalanced ratio of sGAG synthesis and sGAG depletion. Reduced sGAG content eventually results in reduced hydrostatic pressure, compromising the cartilage ability to absorb compressive forces. This could explain why acute or chronic high-intensity loads are described to cause cartilage ECM damage [14], and may explain why healthy rats subjected to strenuous running protocols develop cartilage damage that is closely related to OA onset [15,16].

In summary, moderate biomechanical loads on cartilage can stimulate chondrocyte sGAG synthesis, improve cartilage quality, and protect against OA [7,17], whereas cartilage loading through strenuous running induces OA. In early OA, sGAG levels in cartilage are reduced, making the tissue more vulnerable to damage by mechanical loading. This study investigated whether rat knee joints with sGAG-depleted articular joints through papain injections might benefit from moderate exercise, or whether this increases the susceptibility to cartilage degeneration.

**Figure 1:**
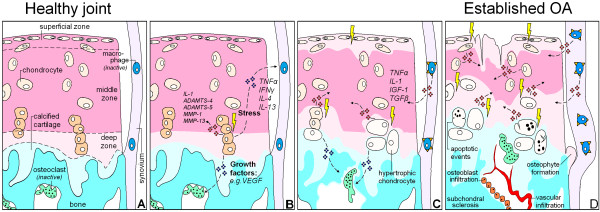
**Hypothetical model that shows in what manner changes of cartilage, subchondral bone, and synovial macrophages all contribute to osteoarthritis development. (A)** Schematically depicted healthy joint with chondrocytes in cartilage extracellular matrix, bone and inactive osteoclasts, and resting synovial macrophages. **(B)** Chondrocytes with a pathological strain produce cytokines and growth factors that diffuse toward the underlying bone marrow and synovium. There these products stimulate osteoclastogenesis and can activate macrophages. **(C)** Progressive phase of OA. Chondrocytes become hypertrophic and produce less sulfated-glycosaminoglycans (sGAGs) to sustain the cartilage, making the ECM more susceptible to compressive forces. Osteoclasts start tunneling through the subchondral bone, which compromises plate stability, and changing its supportive function for the overlying cartilage. Activated synovial macrophages produce growth factors of their own that promote synovial fibrosis, osteophyte formation, and may stimulate ECM degradation. **(D)** Eventually, cartilage is severely sGAG depleted and becomes structurally deprived. Activated macrophages stimulate fibrotic remodeling of the synovium and induce osteophyte growth. Osteoclast activity extends into the calcified cartilage, up to the border with the deep zone of the cartilage. Through subchondral pores, vascular ingrowth occurs into the cartilage. Later, osteoblasts infiltrate and start to deposit bone that results in end-stage sclerosis.

OA is a disease not limited to articular cartilage. OA is considered a ”whole-joint disease” with involvement of subchondral bone, synovium, and articular cartilage changes [18,19] (Figure 1). All of these changes are most likely to play an important role in the complex cascade of pathologic changes during OA development. Therefore, besides measurements on articular cartilage degradation, we also measured subchondral bone changes with μCT and macrophage activation with SPECT/CT. Our results demonstrate that moderate exercise, which does not have an effect on healthy joints, exerts detrimental effects on sGAG-depleted cartilage and also on subchondral bone and synovial macrophage activation.

## Methods

### Study design

Forty 16-week-old male Wistar rats (Charles River Netherlands BV, Maastricht, The Netherlands) were housed in the animal facility of the Erasmus Medical Centre, with a 12-hour light–dark regimen, at 21°C during the experimental period. Animals received standard food pellets and water *ad libitum*.

Intraarticular papain injections were used to reduce cartilage sGAG content in Wistar rat knee joints *in vivo*[20-22]. Previous studies reported three intraarticular injections to induce OA on days 1, 4, and 7 [22,23]. However, after one papain injection, weight bearing of the injected joint is restored to normal 1 week after the injection [24]. Therefore, we injected our animals on days 8, 15, and 22. After they were adapted to the treadmill, with intervals of 1 week to have the rats restore their gait, all animals were injected intraarticularly in their left knee joint with 30 μl papain/L-cystein solution. This solution consisted of 2% wt/vol papain solution (type IV, double crystallized, 15 units/mg; Sigma-Aldrich, St. Louis, MO, USA) and 0.015 *M* L-cystein (Sigma-Aldrich) in saline [22]. Epinephrine (10 μg/ml; Centrafarm, Etten-Leur, The Netherlands) was added to induce vasoconstriction and prevent fast leakage from the knee joint [15,25,26]. All right-knee joints were not injected and served as healthy controls.

Rats were divided into two groups: 20 rats remained sedentary, and 20 rats were forced to run on a motorized treadmill. All running rats were trained to run on a motorized rodent treadmill during the first week (LE-8700; Panlab Harvard Apparatus, Barcelona, Spain) [15]. The following 5 weeks, rats were forced to run for 5 days per week, the first 5 minutes at 20 cm/sec to warm-up, and the following 25 minutes at 35 cm/sec. The pace and duration of this protocol are equal to about 25% of a total exhaustion protocol for rats [27]. In total, running rats covered a total distance of 15 km over the total 6-week period, which is a protocol known to protect from cartilage degradation in both MIA and surgical models for OA [7,9].

**Figure 2:**
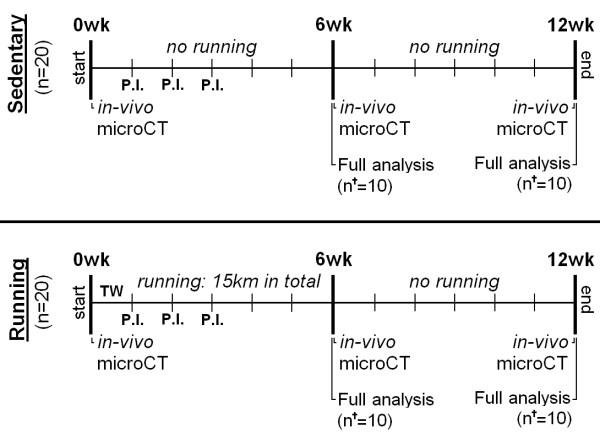
**Experiment design indicating analytic time points and methods for each experimental group.** Forty 16-week-old male Wistar rats received three intraarticular papain injections (P.I.) and divided over two different groups: a sedentary group (*n* = 20) and a running group (*n* = 20). All running rats were subjected to a 6-week moderate running protocol, earlier reported to protect against OA [17]. During the experiment, three μCT scans were made to measure longitudinal subchondral bone changes. At 6 and 12 weeks, a full analysis sequence was done in 10 animals per group (n^†^), consisting of: determination of activated macrophages by using SPECT/CT *in vivo*; and cartilage analysis with equilibrium partitioning of an ionic contrast agent by using (EPIC-)μCT and histology *ex vivo.*

During the study, all animals were longitudinally monitored with μCT to measure subchondral bone changes. At 6 and 12 weeks, 10 rats in both groups were randomly selected for a full analysis sequence. This sequence consisted of SPECT/CT to quantify macrophage activation *in vivo*, and *ex vivo* EPIC-μCT and histology to measure cartilage quality. A detailed planning scheme for all groups and conducted tests is given in Figure 2. The Animal Ethics Committee of the Erasmus Medical Center, Rotterdam, The Netherlands, approved all conducted procedures.

### *In vivo* μCT to measure subchondral bone changes and osteophyte growth

All animals were μCT scanned before start of the running protocol (*t* = 0) and after 6 and 12 weeks of follow-up. In short, under isoflurane anesthesia, after transfer to a holder with the rat in supine position and the hind leg fixed in extension, μCT scans were made by using a Skyscan 1176 *in vivo* μCT scanner (Skyscan, Kontich, Belgium). Ten minutes of scan time were required per knee at an isotropic voxel size of 18 μm, at a voltage of 65 kV, a current of 385 mA, field of view of 35 mm, by using a 1.0-mm aluminum filter, over 198 degrees, with a 0.5-degree rotation step, and a 270-msec exposure time. All scans were performed by using these same settings; all scan data was reconstructed in an identical way, and postprocessing was done as described previously [28-30].

All datasets were segmented with a local threshold algorithm. This algorithm is ideal for accurate bone analysis (3D Calculator software can be requested via email) [31]. With Skyscan analysis software, the tibial epiphysis was selected in the CT scans and analyzed for changes in cortical and trabecular bone. Cortical and trabecular bone were automatically separated by using in-house software [30]. Both subchondral plate thickness (Sb. Pl. Th. in μm) and subchondral plate porosity (Sb. Pl. Por. in mm^3^) of the medial and lateral compartment of the tibial plateau were measured [28]. In the tibial epiphysis, the trabecular thickness (Tb. Th. in μm) and trabecular bone volume fraction (BV/TV), representing the ratio of trabecular bone volume (BV, in mm^3^) to endocortical tissue volume (TV, in mm^3^). Ectopic bone formation (mm^3^) on both lateral borders of the patella was also quantified as a measure for osteophyte growth in these longitudinal μCT scans.

### Determination of activated macrophages by SPECT/CT by using [^111^In]-EC0800

Activated macrophages express the folate-receptor-β [32]. Targeting this folate receptor with folate radioconjugates can be used to monitor activated macrophages *in vivo* by noninvasive nuclear imaging [33,34]. This technique was previously introduced for OA research in a rat model [35]. In brief, phosphate-buffered saline (PBS, pH 6.5) DOTA-Bz-folate (DOTA-Bz-Folate, EC0800, kindly provided by Endocyte Inc., West Lafayette, LA, USA) was incubated with [^111^In]Cl_3_ (Covedien, Petten, The Netherlands) in a mixture of quenchers and sodium acetate (final pH 3.5 to 4) for 15 minutes at 80°C, as described earlier [36]. Quality control was performed with instant thin-layer chromatography medium, by using a silica gel (ITLC-SG) [37,38], and revealed a radiochemical yield of about 93% at a specific activity of 50 MBq/μg.

After radiolabeling, diethylenetriamine-pentaacetic acid (DTPA) was added for complexation of nonincorporated ^111^In. The solution was further diluted in phosphate-buffered saline (PBS) and administered via the tail vein 20 hours before scanning. Each animal received about 55 MBq of [^111^In]-DOTA-Bz-folate under isoflurane anesthesia. SPECT/CT scans were performed with a four-head multiplex multi-pinhole small-animal SPECT/CT camera (NanoSPECT/CT; Bioscan Inc., Washington DC, USA). Each detector head was fitted with a tungsten-based collimator of nine 2.5-mm diameter pinholes; the field of view was 24 mm in width; and energy peaks were set at 170 keV and 240 keV (±10%). All knee joints were scanned with both helical μCT (acquisition time, 5 minutes) and SPECT (acquisition time, 30 min).

After scanning, all datasets were reconstructed at an isotropic CT voxel size of 200 μm and an isotropic SPECT voxel size of 600 μm by using HiSPECT software (Scivis, Göttingen, Germany). All scans were analyzed by using InVivoScope processing software (Bioscan Inc.). A cylindrical region of interest (ROI), based on the CT scan but blinded for the radioactivity, was manually determined for quantification of the radioactivity around and in the knee joint. To correct for the size of the drawn ROI, all data are presented as measured activity (kBq)/mm^3^. To reduce interindividual variation, the absolute difference in measured radioactivity (kBq/mm^3^) of the OA knee joint compared with their internal control joint was calculated. This absolute difference was used when comparing means of papain only and papain combined with running animals.

### Cartilage measurements with EPIC-μCT

Equilibrium partitioning of an ionic contrast agent by using μCT (EPIC-μCT) has a strong correlation with cartilage sulfated-glycosaminoglycan (sGAG) content [39]. sGAG is a key molecule of cartilage, and its content is an indicator of cartilage health [40]. In EPIC-μCT, an equilibrium state exists between sGAG and contrast agent after a 24-hour incubation period. Resulting cartilage X-ray attenuation in these scans is inversely related to sGAG content and thereby represents cartilage quality. This technique is suited for quantitative analysis of cartilage degradation for preclinical evaluation of OA [41].

Animals were euthanized directly after the last SPECT/CT scan, and both knee joints were harvested for EPIC-μCT analysis. The proximal tibial bone was isolated, and soft tissue was removed to a maximal extent, without harming cartilage integrity. Next, all specimens were incubated in 40% solution of ioxaglate (Hexabrix320; Mallinckrodt, Hazelwood, MO, USA), which was diluted in 60% phosphate-buffered saline for 24 hours at room temperature, together with inhibitors of proteolytic enzymes (5 m*M* ethylenediamine tetraacetic acid disodium salt, VWR International, Fontenay, France; and 5 m*M* benzamidine hydrochloride hydrate, Sigma-Aldrich Inc.[42]. EPIC-μCT was performed on the 1176 *in vivo* μCT scanner (Skyscan), by using the following scan settings: isotropic voxel size of 18 μm, a voltage of 65 kV, a current of 385 mA, field of view of 35 mm, a 0.5-mm aluminum filter, 198 degrees with a 0.5-degree rotation step, and a 235-msec exposure time. All scans were performed by using the same settings, and all data were reconstructed identically.

With Skyscan analysis software, these datasets were segmented by using a fixed attenuation threshold between air (30) and subchondral bone (120), selected visually for the best segmentation result in all datasets. In all segmented μCT datasets, ROIs were drawn manually around the cartilage of the medial and lateral plateau of the tibia separately. From these ROIs, the X-ray attenuation (gray values related to sGAG content ranging from 0 to 255) and cartilage thickness (μm) was calculated.

### Histopathologic examination of the knee joint

After EPIC-μCT, the separated parts of the knee joints were fixed in 3.7% phosphate-buffered formaldehyde, decalcified with formic acid, and embedded in paraffin. Sagittal sections of 6 μm thickness were made at 300-μm intervals and stained with Safranin-O with a fast green counterstain to image the amount and distribution of the GAGs. Sections were stained all at once, to minimize artifacts between different samples.

### Statistical analysis

Differences between means of papain-injected and healthy knee joints within the same animal were tested by using paired *t* tests at each time point for all outcome parameters, except when testing differences for osteophyte formation (GraphPad Software, San Diego, CA, USA). Osteophytes did not develop in non-papain-injected control joints in both experimental groups; therefore, we used a one-sample *t* test and tested whether the outcome of the papain-injected joints differed from zero (GraphPad Software). When comparing differences between means of sedentary animals and running animals, unpaired *t* tests were used at each time point for all outcome parameters (GraphPad Software). For all tests, P values < 0.05 were considered statistically significant.

## Results

All sedentary rats increased in weight from 404.3 g (398.8 to 409.6 g) to 454.0 g (445.6 to 462.4 g) after 6 weeks, and further increased to 488.0 g (475.2 to 500.8 g) after 12 weeks. Bodyweight of all running rats at baseline was 416.4 g (411.3 to 421.5 g), during the 6 weeks of treadmill running; this did not increase (mean weight, 408.3 g; 398.2 to 418.3 g) and was significantly lower compared with sedentary rats (*P* < 0.0001). However, during the subsequent 6 weeks of rest, all running rats increased in body weight (mean weight, 485.5 g; 473.0 to 498.0 g) to levels similar to sedentary rats (see Additional file 1: Figure S1).

### Osteoarthritic changes of articular cartilage

**Figure 3:**
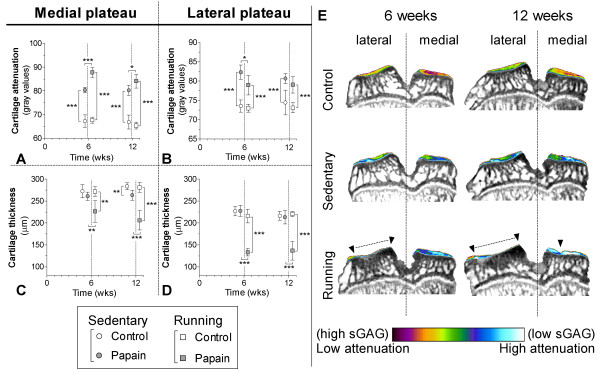
**Cartilage quality and quantity was determined from samples of sedentary (round boxes) and running (square boxes) rats with equilibrium partitioning of a ionic contrast agent by using (EPIC-)μCT (A-D).** The amount of sulfated-glycosaminoglycans (sGAGs) (arbitrary gray values; **A**, **B**) and cartilage thickness (μm; **C**, **D**) were measured of medial **(A,C)** and lateral **(B,D)** cartilage compartments of the tibial plateau harvested from control (blank boxes) and sGAG depleted joints (gray boxes). Attenuation values from EPIC-μCT scans are inversely related to the sGAG content, meaning that a high attenuation corresponds to low sGAG content. Coronal images from representative EPIC-μCT scans of the tibial plateau show the amount of cartilage (erosions indicated with ▼ and dashed lines) and sGAG content (displayed in color) **(E)**. * < 0.05, **P < 0.01, ***P < 0.001; error bars indicate 95% confidence intervals.

As measured with EPIC-μCT, intraarticular injections with papain resulted in sGAG loss from medial and lateral cartilage compartments of the tibia plateau in sedentary animals compared with their control joints (*P* < 0.0001) (Figure 3A,E). Running increased sGAG depletion from cartilage of the medial compartment of papain-injected joints at 6 weeks compared with sedentary papain joints (*P* < 0.0001). At 12 weeks of follow-up, this difference was slightly reduced, but attenuation values were still significantly higher in running animals (*P* = 0.03). Running had no effect on sGAG content in healthy joints. After 6 weeks of running, the thickness of the medial cartilage was slightly lower in the papain-injected compared with their contralateral joint (*P* = 0.007), and compared with sedentary papain joints (*P* = 0.008). Only after 12 weeks, the cartilage in sedentary papain joints slightly degraded and became thinner compared with the contralateral control joint (*P* = 0.004) (Figure 3C,E). The running papain joints, however, showed clear progression of cartilage degradation, and at 12 weeks, remained thinner compared with sedentary papain joints (*P* < 0.0001).

Attenuation values of lateral compartment cartilage were lower in running papain joints compared with papain joints of sedentary animals, indicating a higher sGAG content (*P* = 0.03) (Figure 3B). The lateral cartilage, however, was thinner in running animals (Figure 3D) and showed pronounced denudation of subchondral bone (Figure 3E).

**Figure 4:**
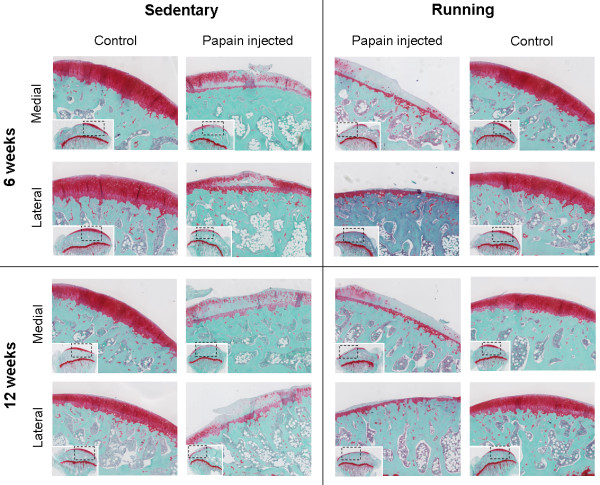
**Histology of safranin-O-stained sagittal sections of medial and lateral tibia plateau cartilage after 6 weeks and 12 weeks of follow-up.** Both in sedentary and in running animals, cartilage of papain-injected joints was severely sulfated-glycosaminoglycan depleted at 6 weeks and 12 weeks. But in running animals, the extracellular matrix showed clear signs of erosion in the medial compartment and even denudation of bone in the lateral compartment.

A similar result was seen on histology sections (Figure 4). These images show that only calcified cartilage remained intact in the lateral compartment of papain-injected joints subjected to running.

### Subchondral bone changes

**Figure 5:**
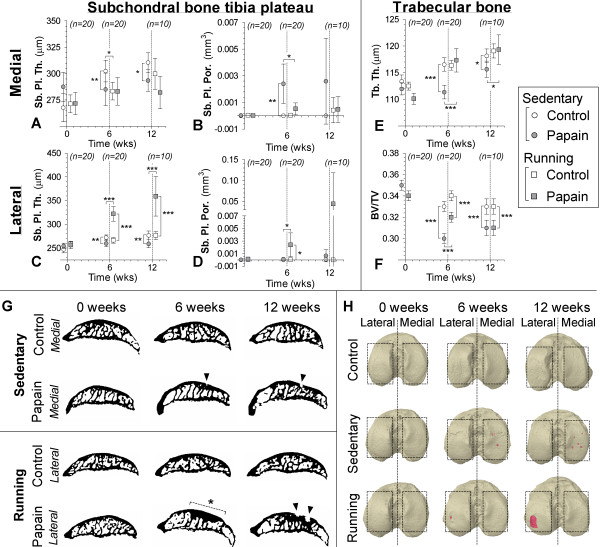
**Subchondral bone changes analyzed with longitudinal *in vivo *μCT in control (white circles) and papain injected (gray squares) knee joints.** Data points were nudged from analyzed time points 0, 6, and 12 weeks for clearer representation of results. Subchondral plate thickness (Sb. Pl. Th.; **A**, **C**) and porosity (Sb. Pl. Por.; **B**, **D**) were measured in the medial **(A,B)** and lateral **(C,D)** compartments of the tibial epiphysis. Changes in trabecular thickness (Tb. Th.; **E**) and trabecular bone volume fraction (BV/TV; **F**) were measured in tibial epiphysis bone marrow. **(G)** Representative sagittal images from binary μCT scans to show most prominent subchondral bone changes, pore development in medial subchondral bone of papain-injected animals (▼), and development of subchondral sclerosis (dashed line and *) in lateral subchondral bone of papain-injected and running animals. Three-dimensional top views of the tibial plateau at different time points **(H)** show subchondral pore (red color) development in papain animals and papain-plus-running animals. *P < 0.05; **P < 0.01; ***P < 0.001; error bars indicate 95% confidence intervals.

*In vivo* μCT showed that subchondral bone of the medial tibia plateau increased in thickness during the experiment in all rats. Subchondral bone thickness in sedentary papain joints (*P* = 0.01) and running papain joints (*P* = 0.01) was slightly lower after 6 weeks of follow-up compared with sedentary control joints (Figure 5A). At 6 weeks, subchondral bone plate pores were detected in only sedentary papain joints (*P* = 0.003 compared with its contralateral joint, *P* = 0.02 compared with running papain joints). In some animals, these pores became larger, but no significant difference was found between sedentary and running animals at 12 weeks (Figure 5B,G,H).

In sedentary animals, lateral subchondral bone thickness showed a response similar to the medial compartment: subchondral bone of sedentary papain joints was thinner compared with its contralateral knee joint (*P* = 0.004 at 6 weeks, and *P* = 0.003 at 12 weeks). However, in papain-injected and running joints, lateral subchondral bone showed a completely different response compared with its medial component. Here severe subchondral sclerosis developed after 6 weeks compared with its contralateral knee joint (*P* < 0.0001), and this sclerotic appearance persisted after subsequent 6 weeks of rest (*P* < 0.0001) (Figure 5C, G,H). In running animals, a clear increase in subchondral plate porosity appeared at 6 weeks compared with their healthy knee joint (*P* = 0.02) and compared to sedentary papain joints (*P* = 0.02). Plate porosity seemed to increase further at 12 weeks, but this was not significant,. probably due to the loss of power, because our group size was reduced to 10 animals at 12 weeks compared with 20 at 6 weeks.

Trabecular bone underlying the subchondral bone plate was thinner in sedentary papain joints compared with its contralateral joint at 6 weeks (*P* < 0.0001), and was still thinner at the end of the experiment (*P* = 0.01). The BV/TV in papain joints of sedentary and running animals showed a clear loss of bone mass throughout the experiment.

**Figure 6:**
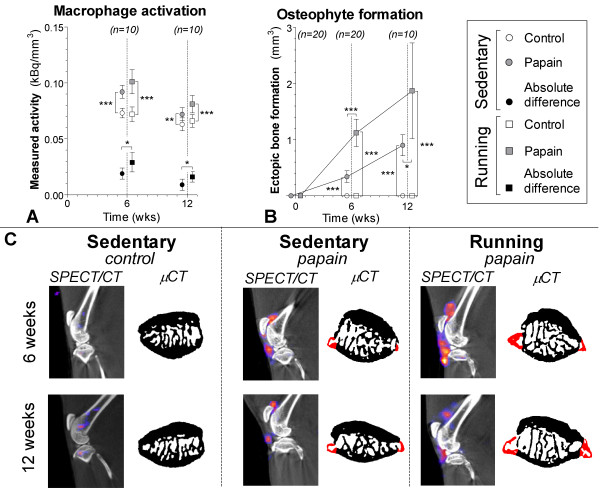
**Macrophage activation determined in sedentary (round boxes) and running (square boxes) rats by injection of [111In]-DOTA-Bz-folate by using SPECT/CT. (A)** Quantitative outcome of measured radioactivity in the control (blank boxes) and papain-injected (gray boxes) knee joints normalized to the size of the cylindrical region of interest (kBq/mm^3^). Absolute differences per animal were calculated (kBq/mm^3^) to reduce interindividual variation (black boxes). A high radioactivity is related to more macrophage activation. **(B)** Ectopic bone formation (mm^3^) as a measure for osteophyte development was quantified on longitudinal bone μCT scans. **(C)** Sagittal SPECT/CT images of knee joints from representative animals. CT images shown in black and white were used for anatomic reference; the SPECT images are shown in color. Transaxial images from patellar bone extracted from binary μCT images show ectopic bone formation (red color). *P < 0.05; **P < 0.01; ***P < 0.001; error bars indicate 95% confidence intervals.

Control joints of all animals in both experimental groups showed no sign of patellar osteophyte formation. In sedentary papain joints and running papain joints, evident patellar ectopic bone formation was noted at 6 (*P* < 0.0001) and 12 weeks (*P* < 0.0001). We also measured a larger volume of ectopic bone formation in papain running joints compared with the sedentary papain joints at both 6 (*P* < 0.0001) and 12 weeks (*P* = 0.03) (Figure 6B,C).

### Macrophage activation

Animals in both groups received 55 ± 2 MBq (mean ± SD) of [^111^In]-DOTA-Bz-folate; no significant difference was found between both experimental groups. Papain joints of sedentary rats showed more macrophage activation measured with DOTA-Bz-Folate SPECT/CT compared with their control knees at 6 weeks (*P* < 0.0001). There was still increased macrophage activation at twelve weeks of follow-up (*P* = 0.02), but the effect was less pronounced. In running animals, macrophage activation was also higher in their papain-injected joints at 6 (*P* < 0.0001) and 12 weeks (*P* = 0.004), but running did not increase macrophage activation in healthy knee joints. We calculated absolute differences between non-papain-injected and papain-injected joints and compared these values between both experimental groups. Both at 6 (*P* = 0.03) and 12 weeks (*P* = 0.03), macrophage activation was higher in papain-injected joints of running than in joints of sedentary animals (Figure 6A,C).

## Discussion

Papain is a proteolytic enzyme that causes chondroitin sulfate release from the cartilage [43]. We confirmed the known effect of intraarticular injections, that papain injections in knee joints of sedentary animals induced sGAG depletion (Figures 3 and 4) [20]. Besides these known changes in cartilage, *in vivo* μCT showed that papain injections induced a loss of medial subchondral plate thickness, formation of subchondral plate pores, and loss of trabecular bone (Figure 5). These aspects were previously related to early OA development [28,30]. Additionally, with *in vivo* folate-receptor-β-targeted SPECT/CT, we found an increased level of activated synovial macrophages in papain-injected joints of sedentary animals (Figure 6).

Cytokine-producing activated synovial macrophages are thought to play a prominent role in osteophyte development [44,45]. Papain-injected joints of sedentary animals also showed clear increased osteophyte formation. In conclusion, papain injections induced prominent sGAG loss from cartilage and showed development of pathologic features in bone and synovium related to early OA progression. However, more tissues in the joint besides articular cartilage might have had damage due to repetitive papain injections. Meniscal cartilage is composed of collagen matrix as well as chondroitin sulfate as its dominant sGAG [46]. Synovium also has sGAG present within its extracellular matrix of the synovial intima and within the interfibrous matrix [47]. Papain injections might also have compromised these structures, but we did not investigate this any further.

In line with other published data, our current study found no negative effects of exercise on healthy joints of running animals compared with healthy joints of sedentary animals (Figure 3) [7,9]. Thus, this exercise protocol can be considered a physiological exposure for healthy sGAG-rich cartilage. Earlier studies reported beneficial effects of moderate physical exercise on healthy cartilage and prevention of osteoarthritis [7,9,10,17]. However, sGAG-depleted cartilage appears highly susceptible for OA progression when exposed to moderate exercise. In running animals, sGAG depletion and cartilage thinning was extensive compared with sedentary controls, and no protective effect of moderate exercise was found. As mentioned before, articular cartilage is likely not the only tissue that had a loss of sGAG due to papain injections. It can be expected that papain induced degradation of meniscal cartilage, ligaments and synovium as well. Possibly, papain induced changes in several joint tissues and may explain why moderate exercise did not protect against articular cartilage degradation, and OA progressed severely in those joints.

Strikingly, a big difference was noted in response between medial and lateral cartilage. Medial cartilage showed a clear loss of sGAG and approximately 25% loss of medial cartilage thickness, whereas within the lateral compartment, subchondral bone was completely denuded of articular cartilage. Healthy cartilage, though, also shows a difference between medial and lateral cartilage, and attenuation values of medial cartilage were about 10% lower compared with lateral cartilage for both sedentary and running animals. This means that medial cartilage is likely to have more sGAG in the medial tibia compartment, which we also found in another study described previously [48]. Rats are known to put more weight on their medial compartment [49]. To withstand these enhanced loads, chondrocytes within the medial compartment might produce higher levels of sGAG. This might be the reason that, after papain injections, more sGAG remained within this compartment compared with lateral tibia cartilage. As a consequence, lateral cartilage was more susceptible for ECM damage, which might have been the reason that lateral cartilage totally eroded.

The enhanced effects of OA in running animals was not limited to cartilage. OA-related pathology in bone and macrophage activation was also enhanced in those joints. Moderate running induced marked changes within the subchondral bone. In the lateral compartment, the subchondral bone plate increased in thickness and led to a sclerotic bone phenotype (Figure 5). We believe that the complete loss of cartilage in this compartment changed the force propagation through the subchondral bone. The sclerosis formation might be an attempt to restore subchondral biomechanical stress levels during the physically active phase, with activation of osteoblasts that increase the subchondral bone mass to adapt to increased physical load exposure [50,51]. Intriguingly, compared with sedentary animals, moderate exercise protected against a loss of trabecular subchondral bone (Figure 5). It is suggested that loss of trabecular bone might be due to unloading of the OA-induced joint because of pain and discomfort for the animals, because gait alterations have been reported in animals with experimentally induced OA [52,53].

Exercise plays an important role in the development and maintenance of bone mass and strength and is known to protect against bone loss in human patients with osteoporosis [54,55]. We believe that exercise was able to exert a similar effect on trabecular subchondral bone maintenance in our experiments.

As mentioned before, during OA development, the macrophages within the synovium become activated [35] and can produce cytokines that enhance osteophyte growth [44] and mediate cartilage destruction [56]. In contrast to joints in sedentary animals, papain-injected joints of running animals had more-pronounced macrophage activation and also larger osteophytes (Figure 6). These results imply that macrophage activation and osteophyte formation are associated. This was previously suggested to be mediated by TGF-β and BMP-2, produced by activated synovial macrophages [44,45]. Depending on stimuli in the environment, macrophages can be activated in different ways. Grossly they can be subdivided into proinflammatory (M1), wound-healing (M2a), and regulatory (M2b) phenotypes, with each subtype characterized by secretion of different cytokines and growth factors [57]. The folate-receptor-β is highly associated with activated macrophages [32,33]. Although this receptor has a slightly higher predisposition on M2 macrophages, both M1 and M2 subtypes do express folate-receptor-β [58]. As a consequence, DOTA-Bz-Folate SPECT/CT cannot address specific subtype macrophage activation. Further *in vivo* studies that modulate macrophage activation and potentially influence folate-targeted SPECT signal are required to provide more insight into the role of macrophage activation and their cytokine production related to OA development.

Numerous animal models exist for OA. Instability models like the anterior cruciate ligament transection (ACLT) model [59], groove model [15,60], the destabilized meniscus model [61], or meniscectomy model [62]) are currently most popular. In our opinion, these models represent an OA etiology related to joint trauma and allows investigation of early changes during OA development. The results from papain-injected and running animals in our study closely resemble end-stage OA-like pathology with pronounced cartilage degradation and subchondral sclerosis. This aspect of severe OA progression in papain-injected joints of running animals might be suited to investigate new treatment strategies designated for regeneration of cartilage in OA joints. This type of OA induction proved to be simple and, in combination with exercise-induced OA in a very reproducible manner with involvement of cartilage, subchondral bone and synovial macrophages.

Additionally, previous reports show that exposure of rat knee joints to low-dose papain induce a reversible sGAG loss from the cartilage [43] and a transient pain response after injection [24]. This suggests that papain does not directly impair chondrocyte viability compared with other chemically induced OA models, like the mono-iodoacetate model that can lead to chondrocyte death shortly after injection [63]. Unimpaired cell viability allows future intervention studies to target chondrocytes therapeutically in an attempt to repair damaged articular cartilage. Although this study did not investigate chondrocytes viability, and more research is necessary to elucidate this feature, experimentally induced OA via papain injections might be a worthwhile contributing model for OA research.

Use of animal models for OA research does not allow direct translation to human patients. It is known that skeletal growth in rats is related to changing cartilage matrix biology and phenotypic characteristics of chondrocytes [64,65]. Therefore, many choices (for example, species, strain, age) related to the study design might have a distinct influence on experimental outcome. With this in mind, our results might still be interesting from a clinical perspective. sGAG is a key molecule for proper cartilage functioning, and the amount of sGAG content is an indicator of cartilage health [40]. Loss of sGAG from articular cartilage is a hallmark of early OA, and it was hypothesized that this occurs well before OA is detected radiographically [66]. For unknown reasons until now, a subgroup of OA patients show signs of rapid OA progression. With radiologic evaluation such as, for example, dGEMRIC to determine cartilage sGAG content, we can investigate whether sGAG depletion and physical exercise may co-induce severe progression in human patients as well. More knowledge about cartilage sGAG content and patient activity level might guide or improve therapeutic interventions as well. Although physical exercise can be beneficial for OA patients [67,68], it might prove to be important that the amount of physical exercise exposure and the intensity level of the activities be carefully balanced in light of their cartilage sGAG status.

## Conclusion

Severe sGAG-depleted cartilage through papain injections is vulnerable for cartilage damage. Moderate physical exercise induced not only rapid OA progression in articular cartilage of papain-injected joints, but involved the whole joint with pronounced subchondral bone adaptation and activation of synovial macrophages.

## Abbreviations

CT: Computed tomography; ECM: extracellular matrix; EPIC-μCT: equilibrium partitioning of an ionic contrast agent by using microCT; MIA: mono-iodoacetate; MMP: matrix metalloproteinase; OA: osteoarthritis; sGAG: sulfated-glycosaminoglycan; SPECT: single-photon-emission computed tomography.

## Competing interests

The authors declare that they have no competing interests. We acknowledge the Dutch Arthritis Association and the BMM/TerM P2.02 Program of the Netherlands Ministry of Economic Affairs and the Netherlands Ministry of Education, Culture, and Science for their financial support.

## Authors’ contributions

Authors MSi, HCG, and MSa contributed to the design of the study, performed the animal experiments, analyzed all data, and drafted the manuscript. SJK, CM, and EB helped with nuclear labeling, SPECT/CT imaging, and revising the manuscript. JHW, CM, GJVMO, MJ, and HW all helped with design of the study, interpretation of data, statistical analysis, and drafting the manuscript. All authors have given their final approval of the version to be published.

## Supplementary Material

Additional file 1: Figure S1 Animal weight. Weight of all animals at 0, 6, and 12 weeks during the study. Nonrunning control animals (open circles) that received papain injections only, increased in weight throughout the study, whereas running animals (shaded boxes) that received papain injections started to increase in weight after the running protocol was completed at 6 weeks. Data points are nudged to prevent overlapping of the data. Click here for file
