# Supplementary figures and images for: Increased physical activity severely induces osteoarthritic changes in knee joints with papain induced sulfate-glycosaminoglycan depleted cartilage
Source: Arthritis Res Ther. 2014 Jan 29;16(1):R32. doi: 10.1186/ar4461 (PMC3978821; doi:10.1186/ar4461)

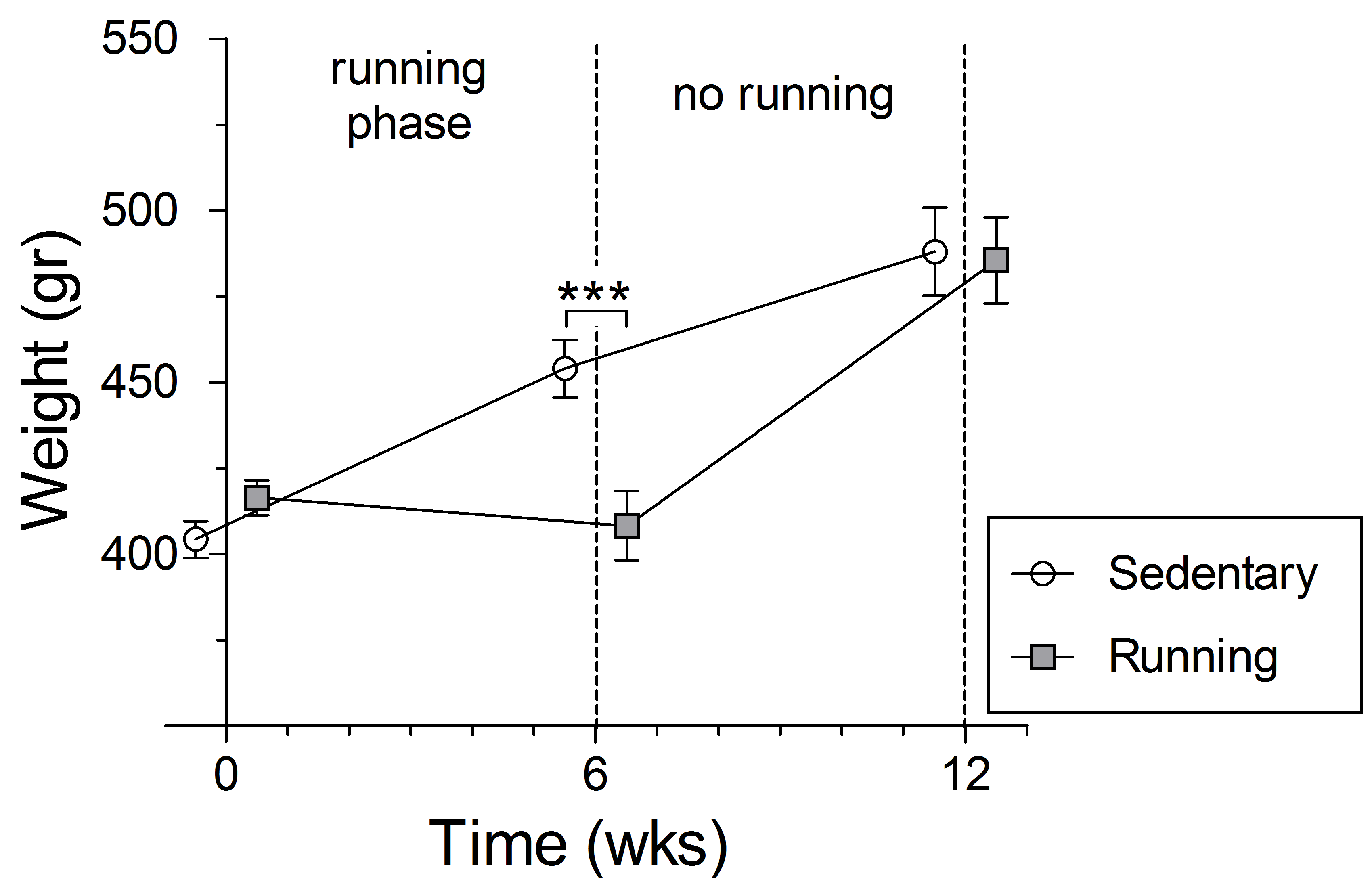

Supplement: Additional file 1: Figure S1 — Animal weight. Weight of all animals at 0, 6, and 12 weeks during the study. Nonrunning control animals (open circles) that received papain injections only, increased in weight throughout the study, whereas running animals (shaded boxes) that received papain injections started to increase in weight after the running protocol was completed at 6 weeks. Data points are nudged to prevent overlapping of the data. [file ar4461-S1.tiff]
